# Entropy: From Thermodynamics to Information Processing

**DOI:** 10.3390/e23101340

**Published:** 2021-10-14

**Authors:** Jordão Natal, Ivonete Ávila, Victor Batista Tsukahara, Marcelo Pinheiro, Carlos Dias Maciel

**Affiliations:** 1 Signal Processing Laboratory, Department of Electrical and Computing Engineering, University of São Paulo (USP), São Carlos 3566-590, Brazil; vhbtsukahara@usp.br; 2 Laboratory of Combustion and Carbon Captur, Department of Energy, School of Engineering, State University of São Paulo (Unesp), São Carlos 3566-590, Brazil; iavila@feg.unesp.br; 3 Versatus Studio, São Carlos 4011-002, Brazil; marcelo@versatus.studio

**Keywords:** entropy, thermodynamics, information theory

## Abstract

Entropy is a concept that emerged in the 19th century. It used to be associated with heat harnessed by a thermal machine to perform work during the Industrial Revolution. However, there was an unprecedented scientific revolution in the 20th century due to one of its most essential innovations, i.e., the information theory, which also encompasses the concept of entropy. Therefore, the following question is naturally raised: “what is the difference, if any, between concepts of entropy in each field of knowledge?” There are misconceptions, as there have been multiple attempts to conciliate the entropy of thermodynamics with that of information theory. Entropy is most commonly defined as “disorder”, although it is not a good analogy since “order” is a subjective human concept, and “disorder” cannot always be obtained from entropy. Therefore, this paper presents a historical background on the evolution of the term “entropy”, and provides mathematical evidence and logical arguments regarding its interconnection in various scientific areas, with the objective of providing a theoretical review and reference material for a broad audience.

## 1. Introduction

Entropy is a measure largely used in science and engineering [1]. It was initially introduced in thermodynamics by Clausius [2], developed by Boltzmann and Gibbs through the 19th century [3] and generalized by Shannon in the 20th century [4] to the point that it can be applied in a broad range of areas. It has been applied to biology [5,6,7,8,9], economics [10,11,12], engineering [13,14,15], linguistics [16,17,18] and cosmology, at the center of one of the greatest open problems in science [10,11,12]. Given this general use in different fields of knowledge, it is important to think about what the measure of entropy actually represents in each different context and the possible equivalence between them.

A misunderstanding about the meaning of entropy is reported in several papers when applied to areas other than physics and information theory [19,20,21,22,23]—sometimes even in these areas—[24,25], and is also reported among students [26]. It is not uncommon to signify entropy as “disorder” [27,28]; although we can often assume for didactic appeal, moderation is necessary so that we do not use a subjective human concept. This is not a rigorous statement since “disorder” is a subjective construction and cannot be measured by entropy [29].

This work intends to contribute with a review of the historical evolution of the concept of entropy, demonstrating the current level of understanding regarding the connection between thermodynamics and information theory. Our target audience is mainly readers outside the fields of physics and engineering, who will have no trouble following two demonstrations about the equivalence between Boltzmann–Gibbs entropy and information theory entropy.

This paper is structured as follows: Section 2 presents the historical evolution of the concept of entropy in physics and information theory; Section 3 explores conceptual relationships between apparitions in these two fields; and finally, we conclude by summarizing the discussed points in Section 4.

## 2. Historical Background

The Industrial Revolution and the development of steam engines was a period of reflection on the physical properties of matter, energy, heat, work and temperature. These phenomena needed to be well understood in order to create efficient engines. It is in this context that the empirical laws that describe the thermal behavior of macroscopic matter were systematized in what it is know today as classical thermodynamics.

In 1825, Sadi Carnot, expanding on his father’s reflections, who had already inferred that perpetual motion was impossible, noted the impossibility of an ideal thermodynamic cycle—Carnot cycle—being reversible [30]. These were actually the first, perhaps rudimentary, formulations of the second law of thermodynamics.

### 2.1. Clausius Entropy

A few decades later, Clausius developed the concept of an extensive quantity, which cannot be measured directly, called entropy. This was associated with an asymmetry in the flow of heat; in nature, heat always flows from a hotter body to a colder one, but the reversal process does not happen spontaneously [31].

The concept of entropy (from the Greek word meaning “change”) was developed to explain the tendency of heat, pressure and density to gradually disappear with time, or similarly, the inevitable generation of heat when work is done on a system by changing temperature. The definition of the state function *S*, in honor of Sadi, called entropy, is as follows:

(1) $$dS=\frac{\delta Q}{T}+\delta S_{gen}$$

with unit J/K. δQ is conventionally used to indicate an inexact differential [32] in which integration depends not only on the starting and ending states, but on the process path in between. On the other hand, entropy is a thermodynamic property; therefore, dS is an exact differential, and integration does not depend on the process path between the starting and ending states. The amount of entropy generation, $\delta S_{gen}$, is null in reversible processes and greater than zero when an irreversible phenomena occurs within the system. However, there is a modification in the system’s entropy due to a change in state, dS, which can be either positive or negative depending on the direction of heat transfer (to or from the system).

For an adiabatic process, δQ=0, and when the entropy differential, dS, is not null, its value is $\delta S_{gen}$ and is always associated with irreversible paths. Contrary to energy, the entropy of an isolated system increases when the process occurs irreversibly, and thus, is not conserved. A reversible process is ideal, but it never really occurs in nature. Therefore, an amount of irreversibility is always present in the system, i.e., the isolated system’s entropy keeps increasing and never reduces.

This concept refers to the increase in entropy principle [33]: the entropy variation of an isolated system (a) never decreases and (b) tends to increase, due to the process’ irreversibility.

### 2.2. Boltzmann–Gibbs Entropy

In the late 1800s, cutting-edge physics was trying to model the ideal gas problem. In this context, Maxwell—and shortly afterwards, Boltzmann—developed the Boltzmann equation as a new model for some problems in classical mechanics, such as that of ideal gas.

**Figure 1:**
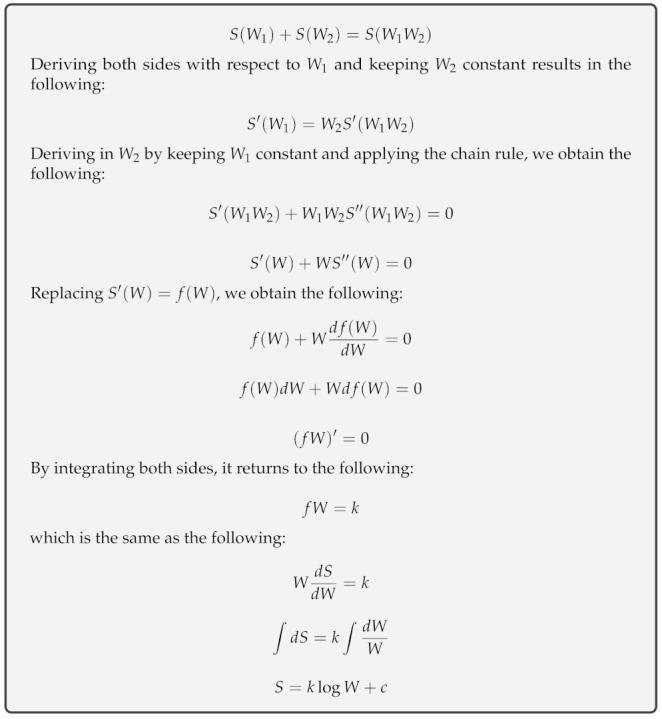
Boltzmann’s entropy formula derivation: since it is known that total entropy *S* is the sum of its parts and the total number of microstates *W* is the product of its parts, the only function S(W) relating these variables is a logarithm.

The entropy, *S*, of an ideal gas is a state function of a possible number of microstates, *W*, for molecules in a macrostate (defined by temperature, volume and pressure). Considering a system comprising an ideal gas and dividing it into two parts, it is hypothesized according to [3] that $S=S_{1}+S_{2}$ and $W=W_{1}\times W_{2}$, given the Boltzmann equation, S=klogW+c, as shown in Figure 1. Hoffmann [34] considered that an ideal gas at 0 K has null entropy and only one microstate, klog1+c=0→c=0, and S=klogW is the entropy of an ideal gas, where k is the Boltzmann constant. Gibbs [35] enhanced the concept of Boltzmann entropy in cases where microstates are not evenly likely:

(2) $$S=-k\sum_{i=1}^{n}p_{i}\log p_{i}$$

where $p_{i}$ is the probability of the i-nth microstate, given that all *W* microstates are evenly likely, and $p_{i}=\left(1,2,3,\ldots ,n\right)=1/n$ and Equation (2) are the same Boltzmann equation. This model led to the notion of entropy with statistical meaning and the conciliation of microscopic reversibility with macroscopic irreversibility.

### 2.3. Shannon Entropy

In 1948, Shannon [4] published the foundational concept of information theory with the concept of entropy of the information of a discrete probability distribution related to the maximum possible data compression.

Following an axiomatic approach, with one enunciate and two desirable properties, it is possible to define the Shannon entropy. Considering an event with *p* probability, and the corresponding function I(p), the two desirable properties are as follows: (i) I(p)>=0 is a decreasing function of *p*; (ii) for any two independent events with probabilities $p_{1}$ and $p_{2}$, $I\left(p_{1}p_{2}\right)=I\left(p_{1}\right)+I\left(p_{2}\right)$. The I(p) interpretation is a measure of “surprise” or “uncertainty” depending on the occurrence of the event. From here, it is possible to determine that the logarithmic function, −logp, satisfies the requested conditions for I(p). Now, let *X* be a random variable. The random variable I(p(X))=−logp(X) is called *self-information* or *information content* of *X* [36].

In the case of a discrete random variable *X* with probability distribution p(x), the average information content about *X* is given by the expected value or Shannon entropy:

(3) $$H\left(X\right)=-\sum_{i=1}^{n}p_{i}\left(x\right)\log p_{i}\left(x\right)$$

The above entropy is dimensionless, although it is common to use the base 2 logarithm and measuring the entropy itself in bits. Apparently, Shannon obtained the name “entropy” from von Neumann himself, as he related [37]:

*“My greatest concern was what to call it. I thought of calling it ‘information’, but the word was overly used, so I decided to call it ‘uncertainty’. When I discussed it with John von Neumann, he had a better idea. Von Neumann told me, ‘You should call it entropy, for two reasons. In the first place your uncertainty function has been used in statistical mechanics under that name, so it already has a name. In the second place, and more important, no one really knows what entropy really is, so in a debate you will always have the advantage".*

Shannon’s original motivation was to create a measure useful in quantifying the channel capacity needed to send a binary message (encoded in a given electrical signal) through telephones lines. One of the uses for the entropy in information theory lies in the measurement of ultimate data compression. For example [1], let us suppose that eight letters, whose frequencies are 1/2, 1/4, 1/8, 1/16, 1/64, 1/64, 1/64 and 1/64, respectively, must be sent. By initially using binary coding, one could assume that 3 bits are needed (000, 001, 010, 011, 100, 101, 110, 111). However, since their frequencies are different, it is possible to encode them as 0, 10, 110, 1110, 111100, 111101, 111110 and 111111, making the average number of bits 2. A fundamental extension of this concept is the derivation of the mutual information between variables *X* and *Y*, given by I(X;Y)=H(X)−H(X|Y), which measures, on average, how much knowing *Y* decreases the uncertainty over *X*.

It is important to emphasize that Shannon entropy by itself does not provide any means to estimate the probability distribution; therefore, it relies on statistics or the observer’s knowledge. In information theory, it is not uncommon to assume uniform distribution, which makes entropy become a trivial function measuring the multiplicity of the different symbols, just like its counterpart measure of Boltzmann–Gibbs entropy that counts the number of possible micro-states of particles in a given volume of space.

### 2.4. Partial Information Decomposition

Recent advances in information theory resulted in the methodology called partial information decomposition [38]. Given a set of variables $R_{1},R_{2},\cdots ,R_{n}$ defined as inputs of a system, and an output *Y*, the objective of this method is to decompose the information on R (be it on the independent R components or joint distributions of these elements). This proposal has the objective of providing information theory with the necessary tools for characterizing the structure of multivariate interactions. Let $A_{1},A_{2},\cdots ,A_{k}$ be nonempty and overlapping sets of R called sources. Since the mutual information for each $I(S;A_{i})$ is an average value over the distributions as mentioned before, two sources might provide the same average amount of information, while also providing information about different outcomes of *S* [38]. Formally, the information about *S* provided by A is given by the following:

(4) $$I\left(S;A\right)=\sum_{s}p\left(s\right)I\left(S=s;A\right)$$

in which the specific information I(S=s;A) is given by the following:

(5) $$I\left(S=s;A\right)=\sum_{a}p\left(a|s\right)\left(\log\frac{1}{p(s)}-\log\frac{1}{p(s|a)}\right)$$

and defining,

(6) $$I_{min}\left(S;A_{1},A_{2},\cdots ,A_{k}\right)=\sum_{s}p\left(s\right)\min_{A_{i}}I\left(S=s;A_{i}\right)$$

the partition information function PI can be defined as follows:

(7) $$I_{\min}\left(S;\alpha \right)=\sum_{\beta ⪯\alpha }PI\left(S;\beta \right)$$

In Equation (7), α belongs to the set of all nonempty subsets of R, and the ordering relationship is given by α⪯β⇔∀B∈β,∃A∈α,A⊆B. The partial information function quantifies the (redundant) information coming from α that does not come from any simpler collection β⪯α.

### 2.5. Algorithmic Information Theory

Algorithmic information theory is the application of elements of Shannon’s theory to algorithms. The most famous of these applications is the Kolmogorov complexity (KC) in a universal Turing machine (a finite state machine that has an input of symbols of a finite alphabet and processes them, returning a new set of symbols) [39]. The KC K(s) of the string *s* is the number of units of information (bits, for example) of the smallest algorithm in a language that can reproduce the object. This measure of complexity has, in its core, an interrogation about randomness. If a string is deterministic, then its KC is low since the code that generates it is simple. For example, the string “001001001001001” and the string “011001101111011” both have 15 bits, but the first one can be coded as “repeat (001) 5 times”, and the second one seems to be random, so the code to generate it will have to contain the entire string.

Shannon’s entropy and KC hold a remarkable relationship. Using the Kraft inequality, it can be shown that the following holds [1]:

(8) $$E\left[\frac{1}{n}K\left(X^{n}|n\right)\right]\to H\left(X\right)$$

and therefore, the compressibility of KC in the universal computer goes to the entropy limit. Moreover, [40] showed that, even though Kolmogorov complexity and Shannon entropy are conceptually different measures, their values are equivalent when dealing with both recursive probability distributions (those which are computable by a Turing machine) or in the case of a time-bounded relationship; this is not always the case in such generalizations as Tsalis and Rényi entropies. However, it is important to notice that these theoretical equivalences suppose that there is perfect knowledge about the distributions originating the data, which is hardly the case [41]; since the KC is distribution independent, which is not the case of the statistical approaches from Shannon’s entropy, one can almost certainly expect a different measurement from these two tools.

New developments in this area resulted in the so-called algorithmic thermodynamics, in which an analogue to the fundamental thermodynamic equation dE=TdS−PdV+μdN and the partition function *Z* are defined in order to study cycles on algorithms analogous to those in heat engines [42], or how problems such as recursion and networks can be dealt with, using information theory tools [43,44].

### 2.6. Algorithmic Information Dynamics

This is a new field focused on the connections between information theory and causality [45]. Algorithmic information dynamics (AID) deals with dynamic systems such as its mathematical model, and is computable, combining perturbation theory and algorithmic information theory, using Bayes’ theorem.

One of the tools used by AID is the coding theorem method (CTM), which deals with compressing without relying on statistical frameworks [43]. It is based on a fundamental identity, given a fundamental prior probability m(s) describing a string and the Kolmogorov complexity K(s): $m\left(s\right)=2^{-K(s)}+c$.

Another tool introduced by AID is the block decomposition method (BDM). One of the motivations justifying both of these methods is the Champernowne constant (x=0.1234567891011…) information content since the sequence generating its digital expansion has no statistical pattern; therefore, it would have maximum entropy on statistical approaches, such as Shannon’s entropy [45].

BDM therefore extends the power of CTM in the field of algorithmic randomness and should be useful in understanding the computation aspects of cognitive processes in the brain [45,46,47].

## 3. Equivalence of Entropy in Thermodynamics and Information Theory

### 3.1. Unity Analysis

The Boltzmann constant linking the thermodynamic macroscopic quantity *S* and the microscopic sum over all the possible micro-states of a system—a dimensionless quantity—clearly has the dimensions of energy divided by temperature (J/K). Since Shannon lacks any proportionality constant, such as the Boltzmann constant, it has no dimension.

Considering purely dimensional units, Shannon’s formulation of entropy seems to have no connection with the formulation of Clausius or Boltzmann–Gibbs entropies. Although being a concept that is purely probabilistic, it shares its randomness nature with the latter. It was demonstrated that the unit is historically associated with the definition of the Kelvin temperature system: the Lagrangian temperature has units of energy in statistical mechanics [48]. In plasma physics, it is common to express temperature in eV [49,50]. In a more generic approach, thermodynamical entropy is dimensionless, and the difference between Shannon and Gibbs’s entropies lies in Boltzmann’s constant.

### 3.2. Underlying Probability

In statistical thermodynamics, the probability of a particular microstate as a function of its energy is given by the so-called Boltzmann distribution, $p_{i}\propto e^{-E_{i}/kT}$, a sufficient and necessary condition for the compatibility of statistical mechanics (with microscopic reversibility) and thermodynamics (with macroscopic irreversibility) formulations and, therefore, the equivalence between the Clausius entropy and Boltzmann–Gibbs entropy.

As we saw earlier, however, in information theory, it is not possible to derive any underlying probability distribution, *which makes Shannon’s entropy a mere combinatorial measure of diversity*. This limitation, so to speak, of Shannon entropy is one of the main attractions of the formulation since it can only quantify meaning when one knows the type of information being treated. Thus, it can be used for a large range of problems involving information.

### 3.3. Shannon Entropy and Thermodynamics

**Figure 2:**
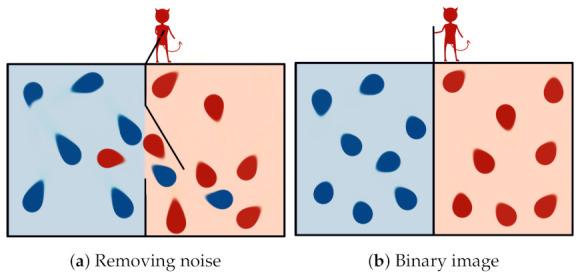
Maxwell’s demon: a being who knows the velocity of every particle in the box and can select their passage, using a opening in the wall that divides it, which could separate those with high energy from those with low energy without performing work, thus violating the second law of thermodynamics. The demon has to forget the past states of the system but, according to Landauer’s principle, this process generates heat (at least kTlog2 J per bit erased) and entropy.

Years before Shannon’s information theory, a thought experiment known as Maxwell’s demon (Figure 2) challenged the second law of thermodynamics. In his own words, it is described as follows [51]:

*“… if we conceive of a being whose faculties are so sharpened that he can follow every molecule in its course, such a being, whose attributes are as essentially finite as our own, would be able to do what is impossible to us. For we have seen that molecules in a vessel full of air at uniform temperature are moving with velocities by no means uniform, though the mean velocity of any great number of them, arbitrarily selected, is almost exactly uniform. Now let us suppose that such a vessel is divided into two portions, A and B, by a division in which there is a small hole, and that a being, who can see the individual molecules, opens and closes this hole, so as to allow only the swifter molecules to pass from A to B, and only the slower molecules to pass from B to A. He will thus, without expenditure of work, raise the temperature of B and lower that of A, in contradiction to the second law of thermodynamics."*

The demon, capable of measuring the kinetic energy of the molecules, can separate fast and slow particles. In this way, the overall entropy of the system will be decreased in a clear violation of the second law of thermodynamics. In addition to that, even in Maxwell’s time, there were already proposals for measurement apparatuses that clearly would not introduce an increase in entropy capable of compensating for the overall decrease proposed in the original setup.

The first important step to clarify the discussion was suggested in 1929 by Szilard [52], introducing a variation of Maxwell setup known as a Szilard engine. The idea was to focus on the measurement itself performed by the demon rather than the work he would have done.

**Figure 3:**
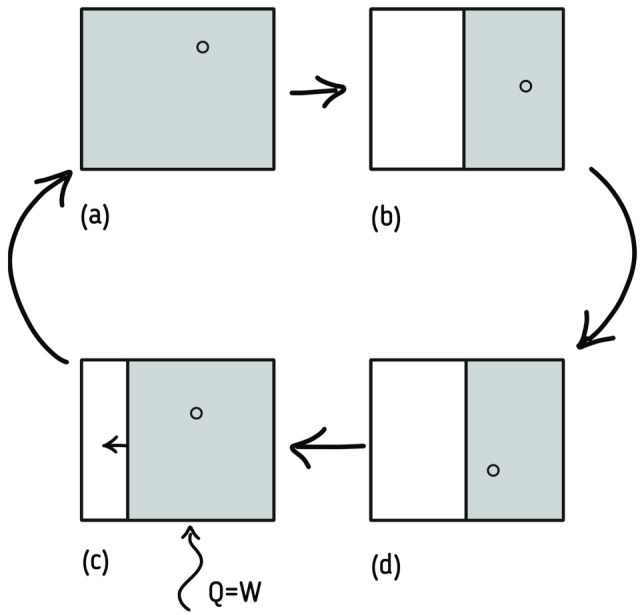
The process of extracting work from a system, thought of by Szilard: in (**a**), there is a single molecule of a fluid inside a box with energy *Q*. If one knows in which half of the box the molecule is (i.e., a single bit of information about its position), a piston can be inserted by halving the box (**b**) and from the fluid expansion, work ((**c**,**d**)) W=Q can be extracted from the system while it returns to its initial state.

The new thought experiment consists of a single molecule of gas inside a box with thermal walls (connected to a reservoir); the demon, in addition to measuring the kinetic energy of the single particle, also inserts and removes a piston in order to divide the vessel in two parts. After its introduction, the gas can isothermally expand to its equilibrium position, doing work that is the equivalent of kTlog2 J (Figure 3). Considering that the demon needs to acquire and store information, even if for a small fraction of time, about the kinetic energy of the particles, it has to be part of the macrostate of the system. Therefore, the information in the demon’s brain can be part of one of the possible configurations, so the second law is not violated.

Although the Szilard engine was the first ever link relating information with thermodynamics, it is still unable to explain the reverse cycle, where the demon forgets what he knew, consequently decreasing the entropy of the system. In fact, the explanation of the reverse cycle came only in 1982 with Bennett, using Landauer’s principle. The principle states that to erase information (logical bit), at least an increment of kTlog2 J of heat is needed [53]. Moreover, the principle can be used to solve the Maxwell’s demon paradox, allowing the demon brain to be updated (forgetting some information to acquire and store others), constituting an irreversible process that generates heat and increases entropy. Rescuing the second law of thermodynamics with the use of information theory also connects Shannon entropy with the already connected entropies of Clausius and Boltzmann–Gibbs. Moreover, the mutual information between the partitions is often null in thermodynamical system since the subsystems are often uncorrelated, which makes the entropy additive in conventional systems; however, in the case of Maxwell’s demon, there is a correlation between the demon and the system, and the solution proposed by Landauer is in accordance with the fluctuation theorem [54].

### 3.4. Information Theoretical Proof that Boltzmann-Gibbs Entropy is the Same as Clausius’s

With the development of information theory in the twentieth century and the concept of maximum entropy for statistical mechanics [55], which states that a system in global and stable thermodynamic equilibrium has reached its maximum entropy by the second law of thermodynamics (being, therefore, in the macrostate that has the most microstates, corresponding to gas velocities), it is possible to derive Clausius’ entropy from Boltzmann–Gibbs formulation of statistical mechanics.

Using Equation (2), and the unitarity principle, $\sum_{i}p_{i}=1$, in which *i* is the i-nth state, we can write the ensemble average energy as follows:

(9) $$\langle E\rangle =\sum_{i}p_{i}E_{i}=U$$

Applying Lagrange multipliers, we have the following:

(10) $$\begin{array}{c} L=-k\sum_{i}p_{i}\log p_{i}-\lambda_{1}\left(1-\sum_{i}p_{i}\right)-\lambda_{2}\left(U-\sum_{i}p_{i}E_{i}\right) \end{array}$$

Differentiating and equaling zero, we have the following:

(11) $$-k\log p_{i}-k+\lambda_{1}+\lambda_{2}E_{i}=0$$

Isolating $p_{i}$, we have the following:

(12) $$p_{i}=\exp\left(\frac{-k+\lambda_{1}+\lambda_{2}E_{i}}{k}\right)$$

Using unitarity with Equation (12), energy can be isolated as follows:

(13) $$\sum_{i}p_{i}=\exp\left(\frac{-k+\lambda_{1}}{k}\right)Z$$

in which *Z* is called the *partition function* and therefore, the following holds:

(14) $$Z=\sum_{i}\exp\left(\frac{\lambda_{2}E_{i}}{k}\right)$$

The partition function combines state functions, such as temperature and energy for the microstates, and has a central role in statistical mechanics [56]. Therefore, using unitarity once more, Equation (13) can be used to isolate $\lambda_{1}$ as follows:

(15) $$\lambda_{1}=k-k\log Z$$

Thus, Equation (12) can be expressed as the following:

(16) $$p_{i}=\frac{1}{Z}\exp\left(\frac{\lambda_{2}E_{i}}{k}\right)$$

Using unitarity again, Equation (12) can be written as follows:

(17) $$\exp\left(\frac{-k+\lambda_{1}}{k}\right)Z=1$$

Therefore, the following holds:

(18) $$\log Z=1-\frac{\lambda_{1}}{k}$$

Rewriting Equation (2) in terms of *Z* results in the following:

(19) $$S=-k\sum_{i}p_{i}\left(\frac{\lambda_{2}}{k}E_{i}-\log Z\right)$$

(20) $$\begin{array}{cc} S & =-\lambda_{2}\sum_{i}p_{i}E_{i}+k\log Z\sum_{i}p_{i}\\ \; & =-\lambda_{2}U+k\log Z \end{array}$$

Using the definition of thermodynamics temperature, we have the following [57]:

(21) $$\frac{1}{T}=\frac{\partial S}{\partial U}$$

Since $\frac{\partial S}{\partial U}=-\lambda_{2}$, Equation (2) can be written as follows:

(22) $$S=\frac{U}{T}+k\log Z$$

Now, let us change the system energy by an inexact differential δQ. Each microstate increases its energy by $q_{i}$. A calculation of the change in entropy results in the following:

(23) $$dS=\frac{\delta U}{T}+k\delta \log Z$$

Calculating the second term, we have the following:

(24) $$\delta \log Z=\frac{d\log Z}{dZ}\delta Z=\frac{\delta Z}{Z}$$

Considering that $Z=\sum_{i}\exp\left(-E_{i}/kT\right)$, the new partition function can be written as follows:

(25) $$Z=\sum_{i}\exp\left(-\frac{E_{i}+q_{i}}{kT}\right)$$

Applying Taylor expansion in $e^{-q_{i}/kT}$, since $q_{i}$ is infinitesimal, a good approximation is the following:

(26) $$\exp\left(-\frac{q_{i}}{kT}\right)=1-\frac{q_{i}}{kT}$$

Therefore, this new partition function can be written as follows:

(27) $$Z=\sum_{i}\left(1-\frac{q_{i}}{kT}\right)\exp\left(-\frac{E_{i}}{kT}\right)=Z_{0}+\delta Z$$

Therefore, the partition function variation is given by the following:

(28) $$\delta Z=-\frac{1}{kT}\sum_{i}q_{i}\exp\left(-\frac{E_{i}}{kT}\right)$$

According to the first law of thermodynamics, the change in *U* can be expressed as follows:

(29) $$\delta U=\sum_{i}\delta E_{i}p_{i}+\sum_{i}q_{i}p_{i}=\delta Q+\delta W$$

Calculating δlogZ, replacing (28) in (24), we have the following:

(30) $$\delta \log Z=\frac{-\frac{1}{kT}\sum_{i}q_{i}\exp\left(-\frac{E_{i}}{kT}\right)}{Z}$$

However, through Equations (16) and (21), it is known that the following holds:

(31) $$p_{i}=\frac{1}{Z}\exp\left(-\frac{E_{i}}{kT}\right)$$

and therefore, we have the following:

(32) $$\delta \log Z=-\frac{1}{kT}\sum_{i}p_{i}q_{i}$$

This value is exactly −δW/kT. By replacing this relation in Equation (23), we obtain the following:

(33) $$dS=\frac{\delta Q}{T}$$

which is the Clausius first definition of entropy.

### 3.5. Using Kullback–Leibler Divergence to Obtain an Analogous of the Second Law of Thermodynamics

Today, modern supervised machine learning techniques use extensively a measure formulated using the Kullback–Leibler divergence as a cost function when training classifiers, the cross-entropy. It is important to show the connection between this important measure of information theory with the second law of thermodynamics.

The relative entropy or Kullback–Leibler divergence between two probability distributions over *X*, p(x) and q(x) is defined as follows:

(34) $$D\left(p||q\right)=\sum_{x\in X}p\left(x\right)\log\frac{p(x)}{q(x)}$$

It should be noticed that D(p||q)=0 if p=q (considering 0log0/0=0) in Equation (34). However, it is not a distance in a formal sense since D(p||q)≠D(q||p). Relative entropy measures how similar the two distributions are.

Let us assume that $\alpha_{n}$ and $\alpha_{n}^{\prime }$ are distributions of states in the Markov chain state space describing a physical thermal system. Once $\alpha_{n+1}$ and $\alpha_{n+1}^{\prime }$ are their evolution in time, and *p* and *q* are their corresponding joint distribution, and given that they are in the Markov chain space, we can write the following:

(35) $$p\left(x_{n},x_{n+1}\right)=p\left(x_{n}\right)\pi \left(x_{n+1}|x_{n}\right)$$

(36) $$q\left(x_{n},x_{n+1}\right)=q\left(x_{n}\right)\pi \left(x_{n+1}|x_{n}\right)$$

in which *r* is the probability transition in the Markov chain. Two relations can be obtained for these equations:

(37) $$\begin{array}{c} D(p\left(x_{n},x_{n+1}\right)||q\left(x_{n},x_{n+1}\right))=D(p\left(x_{n}\right)||q\left(x_{n}\right))+D(p\left(x_{n+1}|x_{n}\right)||q\left(x_{n+1}|x_{n}\right)) \end{array}$$

(38) $$\begin{array}{c} D(p\left(x_{n},x_{n+1}\right)||q\left(x_{n},x_{n+1}\right))=D(p\left(x_{n+1}\right)||q\left(x_{n+1}\right))+D(p\left(x_{n}|x_{n+1}\right)||q\left(x_{n}|x_{n+1}\right)) \end{array}$$

Due to the fact that both *p* and *q* come from the Markov chain, we have $p\left(x_{n+1}|x_{n}\right)=q\left(x_{n+1}|x_{n}\right)=\pi \left(x_{n+1}|x_{n}\right)$, $D(p\left(x_{n+1}|x_{n}\right)||q\left(x_{n+1}|x_{n}\right))=0$. Since relative entropy is always non-negative, we have the following:

(39) $$D(p\left(x_{n}\right)||q\left(x_{n}\right))\geq D(p\left(x_{n+1}\right)||q\left(x_{n+1}\right))$$

(40) $$D(\alpha_{n}\left|\right|\alpha_{n}^{\prime })=D(\alpha_{n+1}\left|\right|\alpha_{n+1}^{\prime })$$

This means that, as time passes, the probability distributions in the Markov chain (and therefore, in the system being described) becomes increasingly similar. $D(\alpha_{n}||\mu )$ generates a monotonically decreasing sequence and has a limit. Assuming that $\alpha_{n}^{\prime }=\mu$ is a stationary distribution over time, $\alpha_{n+1}^{\prime }=\mu$. Hence, we have the following:

(41) $$D(\alpha_{n}\left||\mu \right)\geq D\left(\alpha_{n+1}|\mu \right),$$

which means that each distribution becomes closer to stationary as time passes. In thermodynamics, a stationary distribution is considered uniform with *W* different states. By applying Equation (34) in Equation (41), we have the following:

(42) $$D(\alpha_{n}\left||\mu \right)=\log W-H\left(\alpha_{n}\right)=\log W-H\left(X_{n}\right)$$

Since $D(\alpha_{n}||\mu )$ decreases, $H(X_{n})$ must increase as time passes.

## 4. Conclusions

The concept of entropy started as an abstract mathematical property in thermodynamics at the center of the first Industrial Revolution. It developed with the advent of statistical mechanics in an important measure with a mathematical formulation that later would become ubiquitous. Further development came from information theory with Shannon entropy, which is just a combinatorial diversity, being compatible with Boltzmann–Gibbs entropy under certain conditions. Even more recent developments clarified that information is not something amorphous; instead, a medium is needed in order to be acquired and stored. Hence, the medium is the connection between temperature and the bit of information—the connection between thermodynamics and information theory, at least on a macroscopic scale, in which the thermodynamics entropy is additive since the correlation between parts of a system is null; otherwise, a more precise description, involving the fluctuation theorem is necessary. Finally, important concepts related to Shannon entropy seem to be at the center of the fourth industrial revolution [41].

It is worth noting that in the context of Shannon entropy, which applies to any probability distribution, the Boltzmann distribution is only a special case. The possibility of choosing different distributions makes this formulation applicable to several domains, but it is imperative to keep the application context in mind in order to understand the meaning of the measures. 

## Data Availability

Since his is a theoretical paper there is no data to be available.
